# The Role of Biomarkers in Diagnosis of Sepsis and Acute Kidney Injury

**DOI:** 10.3390/biomedicines12050931

**Published:** 2024-04-23

**Authors:** Gillene Santos Ferreira, Melissa Lopes Frota, Maria José Dias Gonzaga, Maria de Fátima Fernandes Vattimo, Camila Lima

**Affiliations:** Department of Medical-Surgical Nursing, School of Nursing, University of São Paulo, São Paulo 05403-000, Brazil; gillene.fsantos@usp.br (G.S.F.); melissalopesfrota@hotmail.com (M.L.F.); mariadias@usp.br (M.J.D.G.); nephron@usp.br (M.d.F.F.V.)

**Keywords:** sepsis, acute kidney injury, biomarkers, neutrophil gelatinase-associated lipocalin, cell-free DNA

## Abstract

Sepsis and acute kidney injury (AKI) are two major public health concerns that contribute significantly to illness and death worldwide. Early diagnosis and prompt treatment are essential for achieving the best possible outcomes. To date, there are no specific clinical, imaging, or biochemical indicators available to diagnose sepsis, and diagnosis of AKI based on the KDIGO criterion has limitations. To improve the diagnostic process for sepsis and AKI, it is essential to continually evolve our understanding of these conditions. Delays in diagnosis and appropriate treatment can have serious consequences. Sepsis and AKI often occur together, and patients with kidney dysfunction are more prone to developing sepsis. Therefore, identifying potential biomarkers for both conditions is crucial. In this review, we talk about the main biomarkers that evolve the diagnostic of sepsis and AKI, namely neutrophil gelatinase-associated lipocalin (NGAL), proenkephalin (PENK), and cell-free DNA.

## 1. Introduction

Sepsis remains a pressing global health concern despite medical advances, according to the World Health Organization (WHO), due to its high incidence, prevalence, and mortality rates [1,2,3]. As estimated, there have been 48.9 million sepsis cases worldwide, causing 11 million deaths, which accounts for 19.7% of global mortality [4,5]. The absolute mortality rate can reach one-third to one-sixth of all affected patients, respectively. Sepsis is also one of the most expensive complications in hospital healthcare, contributing to 6.2% of total hospitalization costs or approximately $23 billion annually [5,6].

Acute kidney injury (AKI) is a common complication associated with high costs and significant morbidity and mortality [7,8]. The heterogeneity of AKI, as in sepsis, makes diagnosis and treatment difficult [9]. This clinical syndrome complicates the course and worsens the outcome in a significant number of patients, both in hospital and community settings [10,11]. The global burden of AKI-related mortality far exceeds that of breast cancer, heart failure, or diabetes, with an estimated 2 million people dying from AKI every year [12,13,14].

The multifaceted origin of organic dysfunction in sepsis results in diverse signs and symptoms, often with subtle and nonspecific clinical manifestations [15]. Additionally, the actual diagnosis of AKI is late and does not provide information about the cause, type, and severity of AKI [16]. Early diagnosis and prompt treatment are crucial for achieving the best possible outcomes. Therefore, it is essential to continually evolve the diagnostic process.

### 1.1. Diagnosis of Sepsis

The concept of sepsis has changed significantly over time, as have its criteria. In 1991, sepsis was first defined as a disease called Systemic Inflammatory Response Syndrome (SIRS), due to a suspected or confirmed infection with two or more organic dysfunctions. These dysfunctions can include increased heart and respiratory rate, temperature, leukopenia, or leukocytosis. During the same period, septic shock was described as hypotension and persistent organ dysfunction, even with the use of volume and the need for vasopressor administration [17].

In 2001, even recognizing the limitations, the existing definitions were updated with clinical and laboratory variables, resulting in Sepsis-2.0 [18]. Neither Sepsis1.0 nor -2.0 on their own indicate a complication course. In 2016, the Third International Consensus Definitions for Sepsis and Septic Shock (Sepsis-3.0) recommended the use of Sequential Organ Failure Assessment (SOFA) and was validated in large cohorts from large retrospective databases in the USA and Germany [19,20]. The most recent definitions are: sepsis is a potentially fatal organ dysfunction caused by a dysregulated immune response to an infection, which can be caused by viruses, fungi, bacteria, or protozoa, and septic shock occurs in sepsis with hypotension that is not responsive to fluid resuscitation, where the use of vasopressors is required to maintain a mean arterial pressure (MAP) of 65 mmHg or higher and a serum lactate level equal to or above 18 mg/dL (2 mmol/L) [20].

The validation of sepsis diagnosis is defined by a variation of two or more points in the SOFA score in the presence of an infection. The SOFA score was developed by the European Society of Intensive Care Medicine as a method to describe individual organ dysfunction/failure. The main dysfunctions include neurological, respiratory, cardiovascular, gastrointestinal, renal, hematological, and endocrinological, and they reflect reduced oxygen supply and/or cellular changes. In this assessment, each variable is scored from 0 to 4 [21]. 

One of the key points in sepsis, besides early diagnosis, is the therapeutic target definition. Even when the infectious focus is known, the etiological agent and its resistance are often unknown. Blood cultures have low sensitivity, frequently leading to missed identification of all causative organisms. Therefore, advances in molecular diagnosis of sepsis have recently been explored for the rapid identification of pathogens [22]. 

### 1.2. Diagnosis of Acute Kidney Injury

The concept of sepsis has changed significantly over time, as have its criteria. Similarly, the diagnosis of AKI has evolved over time, being a syndrome characterized by an abrupt drop in the glomerular filtration rate, primarily based on the increase in serum creatinine and the reduction in urinary output.

The Acute Dialysis Quality Initiative (ADQI) proposed, in 2004, a classification based on the severity of renal injury, known as Risk, Injury, Loss End-Stage Renal Disease (RIFLE). In 2007, there was a revision of RIFLE, and the Acute Kidney Injury Network (AKIN) suggested adding the criterion of an increase in serum creatinine = >0.3 mg/dL in 48 h to define AKI. In 2012, the Kidney Disease Improving Global Outcomes (KDIGO) incorporated criteria from the RIFLE and AKIN classifications (see Table 1) [23]. The new AKI classification determines that:

The standardization of the AKI diagnosis has allowed us to compare diagnoses made in different settings. However, early diagnosis remains a challenge. First, there are limitations regarding the use of serum creatinine and urinary output. Second, determining the need for renal replacement therapy is difficult due to a lack of information about whether the AKI is transient or persistent. Lastly, advances in research identifying early biomarkers have so far been inaccessible in clinical practice [12].

### 1.3. The Crosstalk between Acute Kidney Injury and Sepsis

The kidney is one of the earliest injured organs during sepsis. AKI develops in about two-thirds of patients with septic shock [13,24]. Therefore, it is reasonable to consider AKI as an early sign of sepsis, and sepsis is one of the most important etiologies of AKI in this setting. Sepsis-associated acute kidney injury (SA-AKI) is common in critically ill patients and is strongly associated with adverse outcomes, including an increased risk of chronic kidney disease, cardiovascular events, and death. The pathophysiology of SA-AKI remains undefined, although microcirculatory dysfunction, cellular metabolic reprogramming, and dysregulated inflammatory responses have been implicated in preclinical studies [13].

Recently, the consensus reported that SA-AKI is defined as the occurrence of acute kidney injury within 7 days of the onset of sepsis, diagnosed according to the criteria of the Kidney Disease Improving Global Outcome and Sepsis 3 [25].

Sepsis is a complex condition that involves a disruption of the body’s immune response to an infection. It is characterized by a dysregulated balance of inflammation and anti-inflammation.

The pathophysiology of sepsis and AKI are intrinsically interrelated. Various mechanisms can contribute to injury in SA-AKI, such as systemic and renal inflammation, complement activation, RAAS dysregulation, mitochondrial dysfunction, metabolic reprogramming, microcirculatory dysfunction, and macrocirculatory abnormalities [25]. In septic conditions, the SIRS process, caused by infection, is related to the release of systemic pro-inflammatory factors, which causes endothelial damage, microvascular dysfunction, deficits in tissue oxygenation, and, consequently, organ damage [26]. The nephron, the functional unit of the kidney, is predominantly composed of capillaries, which therefore highlights the vulnerability that this organ presents to these mechanisms [26].

On the other hand, a UTI, which, depending on its severity, can progress to AKI, is the third infectious cause most related to progression to a septic condition, behind lung and abdominal infections [27], triggering the pathophysiological condition described previously [26]. Furthermore, patients who progress with severity may require mechanical ventilation and administration of nephrotoxic drugs, which tend to increase harmful effects on renal function [28], so it is essential to explore new biomarkers, which can allow the application of interventions, evaluate responses to treatment, detect biological abnormalities, and assess the prognosis according to the development of the disease [29]. The 28th Acute Disease Quality Initiative consensus reported that SA-AKI recommends utilizing sepsis biomarkers in conjunction with functional and tubular injury-related biomarkers to enhance the prognosis of early or late SA-AKI [25].

## 2. Discussion

The role of biomarkers in the detection of sepsis and AKI is fundamental for the diagnosis, treatment, and prognosis of the disease. However, so far, no single biomarker has shown to have sufficient specificity or discriminatory value to be used unequivocally and reliably in the diagnosis and/or prognosis of these pathologies. We have listed the potential BMs used in both pathologies to assess whether it is possible to diagnose sepsis in AKI and vice versa.

The combination of biomarkers involved in different pathways related to sepsis and AKI, along with clinical information, can be particularly useful in the diagnosis or risk stratification for patients with sepsis and AKI.

We have selected three promising biomarkers for the diagnosis of AKI and sepsis: NGAL, PENK, and cfDNA. Their pathophysiological association with both conditions and the main confounding factors are summarized in Table 2 and will be detailed in the following sections.

The use of biomarkers can help overcome the limitations of current diagnostic standards for AKI and sepsis. In AKI diagnosis based on the increase in serum creatinine (Scr) and/or decrease in urine output, the main limitations of Scr include delay (its increase occurs only after 48 h of injury), underestimation (fluid overload), decreased production in sepsis, and, value dependence on factors influenced by muscle mass structure and other factors [30]. The measurement of urine volume in diapers is unreliable, and the use of urinary catheters for more accurate monitoring can lead to infectious complications. Additionally, even in oliguric cases, urine volume may not be indicative of renal dysfunction, as a significant percentage of cases do not exhibit oliguria (25% to 80%) [30].

In the case of sepsis, the limitations are more extensive as there is currently no standard biological marker for diagnosis. In clinical practice, procalcitonin, lactate, and CRP can be helpful. The limitations of procalcitonin include elevation in other conditions such as trauma, burns, cardiogenic shock, major surgeries, and renal injury [31]. Moreover, the specificity of procalcitonin for diagnosing sepsis is relatively low. Some patients do not exhibit an increase in procalcitonin even in cases of septic shock, and there may be a delay in its elevation after the onset of sepsis [32]. Lactate elevation, seen in perfusion abnormalities and oxidative metabolism impairment, is not a reliable early indicator of sepsis and lacks specificity [33], and C-reactive protein (CRP) lacks specificity for bacterial infection and is seen to be raised in most other causes of inflammation. 

**Figure 1:**
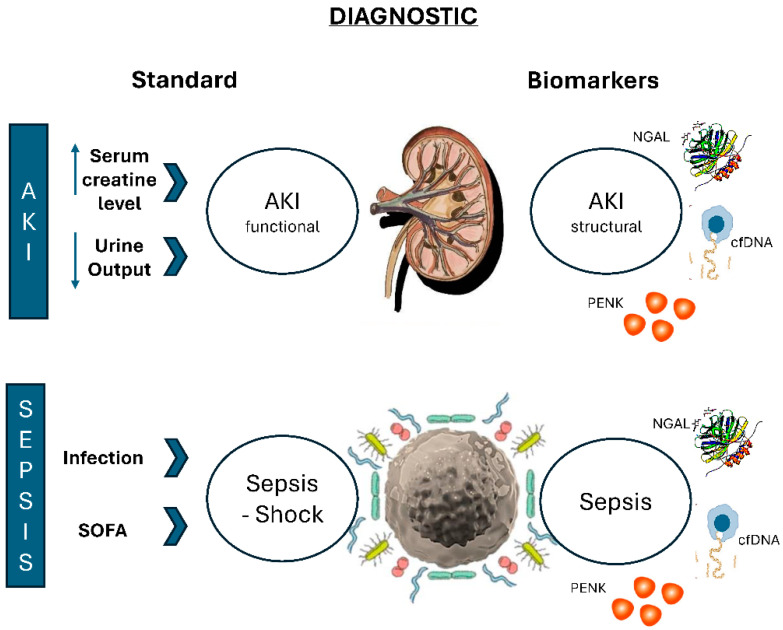
Current Diagnostic Approaches and Future Clinical Applications of Biomarkers in Sepsis and Acute Kidney Injury (AKI). Legend: The figure aims to exemplify the current diagnostic methods for both sepsis and acute kidney injury (AKI) and their potential future clinical application using biomarkers. The diagnosis of acute kidney injury (AKI) is based on the increase in serum creatinine and/or the decrease in urine output according to the KDIGO criteria. The main limitation of the current diagnosis lies in the progression of the disease to more severe stages, where functional injury is already present; biomarkers could diagnose structural damage before functional injury occurs. This early identification would allow for the implementation of targeted therapies, altering the natural course of the disease. On the other hand, the diagnosis of sepsis is based on suspicion or evidence of infection, coupled with organ dysfunction assessed by the SOFA scale with a score greater than two (Sepsis 3.0). The primary limitation of the current diagnosis is the progression of the disease to more severe stages, such as septic shock, where clinical severity can be fatal regardless of therapeutic interventions; thus, once again, biomarkers become useful in early detection and as a therapeutic target with better chances of recovery.

Although still in the research phase, the hypothesis is that in clinical practice, they could assist in earlier diagnosis in both conditions, providing more detailed information such as the location/type of injury in the case of AKI or the site/type of infection in sepsis (Figure 1). The combined use of these biomarkers could enhance diagnostic accuracy, given the infinite heterogeneity present in both conditions. For example, AKI can stem from various causes such as pre-renal, renal, and post-renal factors, including ischemic, nephrotoxic, and obstructive origins, among others. Therefore, a panel of biomarkers may provide a more comprehensive assessment than a single biomarker alone to better understand the global kidney dimension and the various pathways related to AKI. Similarly, in sepsis, the potential pathophysiological pathways differ among the three biomarkers listed in Table 2. Recognizing these distinctions can not only aid in risk stratification and early detection but also improve disease management by offering a more refined understanding of its complexities and directing the use of antibiotics more effectively towards the infecting pathogen.

### 2.1. Neutrophil Gelatinase-Associated Lipocalin—NGAL

Neutrophil gelatinase-associated lipocalin (NGAL) can currently be considered the main diagnostic biomarker in AKI and sepsis. Its role as a biomarker has been recognized in several clinical studies. In the study by Kjeldsen et al. in 1993, NGAL was created from the isolation of a lipocalin as a protease-resistant polypeptide, covalently linked to neutrophil gelatinase, also named as siderocalin, lipocalin 2, or oncogene 24p [34]. NGAL is a small protein molecule that is 25 kilodaltons (kDa) covalently linked to gelatinase in specific neutrophil granules. The levels of NGAL found in human tissues, including the kidneys, stomach, lungs, trachea, and colon (adult and fetal), are signaled at negligible levels [35]. Furthermore, NGAL has anti-inflammatory characteristics which are validated by increased expression in proliferative epithelia, regions of inflammation and intestinal malignancy [36].

The manifestation of NGAL is expressed both in normal kidneys, in which it is released by the thick ascending limb and by the interspersed cells of the espresso collecting duct, and in the proximal tubular epithelium, since filtration occurs by the glomerulus and is reassimilated by the proximal tubule in a dependent manner, involving megalin [36,37]. The physiological function of NGAL in the kidneys is still unknown; however, there is a scientific belief that NGAL acts in renal morphogenesis [38]. NGAL apparently plays a predominant role in cell proliferation, both in repair processes and in tubular re-epithelialization. This biomarker is an additional iron transport pathway, where the increase in transcription of heme oxygenase is determined, an enzyme that acts in the protection and preservation of proximal tubular cells, with proliferative and antiapoptotic action [39,40].

Conditions that may interfere with NGAL to be used as a biomarker are sepsis, chronic obstructive pulmonary disease, cardiac dysfunction, diabetes, and hypertension. These factors can impair the measurements of NGAL levels present in the body [41]. Age apparently is an influencing factor in NGAL rates, as in children the levels are higher when compared to older patients; gender is another factor, since female patients have a higher predictive value compared to males; and the other factors are urinary tract infection and impaired renal function, with greater predictive value in patients with chronic kidney disease [42,43,44].

Among the various biological functions that have been proposed for NGAL, however, specifically in the kidneys, the release of NGAL is correlated with nephrotoxic and schematic insults. Another situation that contributes to an extra increase in urinary NGAL concentration is the decrease in tubular reabsorption after AKI, causing a status similar to that of kidney troponin [42,43,44,45].

In a study, NGAL was analyzed in several contexts, both in pediatric and adult patients who underwent cardiac surgery, patients with serious illnesses, kidney transplants, and emergency rooms [46,47]. A study by Constantin et al. highlighted the potential of NGAL in diagnosing AKI, showing a sensitivity of 82%, specificity of 97%, and receiver operating characteristic (ROC) area under the curve (AUC) of 0.92 for predicting AKI [48]. In another multicenter study, Di Somma et al. concluded that the serial assessment of NGAL at different times enabled the ability to rule out AKI within six hours after the start of patient care in the emergency room [49]. Haase et al. and Zhou et al., in their meta-analyses, highlighted the prognostic and diagnostic value of NGAL for AKI, demonstrating extreme sensitivity and assertiveness in the diagnosis of AKI [50]. Urinary NGAL assists in the classification and stratification of patients with established AKI, with the ability to predict the worsening of RIFLE class outcomes, such as in-hospital mortality and renal replacement therapy [51]. In addition to AKI, NGAL can be used to predict adverse outcomes in the medium term, since its early measurement in the context of critical situations disease can identify patients with AKI at increased risk of death or progression to CKD in the following nine months [8]. Another study also demonstrated the potential ability of this biomarker to detect and predict AKI; however, in the long term, there were no considerable clinical differences [52].

These conclusions highlight the potential of NGAL as an essential biomarker in the diagnosis of AKI, but due to its variability in platforms and cut-off points, difficulties are encountered in generalizing it for a more comprehensive use in clinical practice. The platform favored the understanding of the different types of NGAL present, such as the 25 kDA NGAL monomer synthesized by monocyte tubular epithelial cells, the 45 kDA NGAL homodimer secreted by neutrophils covalently complexed with metalloproteinase-9 (MMP9). This can contribute to the maturation of understanding about the diagnosis of sepsis. The 25 kDA NGAL found in tubular epithelial cells may be more appropriate in the diagnosis of AKI. In turn, the test that uses the 45 kDA homodimer secreted, for the most part, by neutrophils is more commonly used in the diagnosis of sepsis [45].

Therefore, the ability of NGAL to reflect neutrophil activation, which is common in sepsis, indicates that NGAL may play an important role in the early diagnosis and stratification of septic patients. Studies have shown that measuring NGAL can help locate septic patients and predict the severity of the disease, making it a promising field of research in the context of diagnosis and management of sepsis.

NGAL associated with Fetuin-A showed good efficacy in predicting 28-day mortality in septic patients [51]. Another study of 100 septic patients, 37 of whom had bacterial-type sepsis, found the standard plasma NGAL cut-off value for predicting sepsis to be 570 ng/mL, AUC 0.69. The use of NGAL > 570 ng/mL with scores (diabetes mellitus, presence of chills, rapid sequential organ failure assessment (qSOFA) score > 2 and clear focus of infection) > 7 was predominantly suggestive of bacterial infection with the interval likelihood ratio (LR) of 7.77 [53]. In a neonatal population, a similar cut-off value of 455 μg/L (sensitivity 71.4%, specificity 100%) and 1104 μg/L (sensitivity 39.3%, specificity 95.2%) was found to anticipate sepsis and non-survival, respectively [54]. Another study found a lower cut-off point for diagnosing sepsis, 250 ng/mL, sNGAL-0 h to predict 28-day mortality with a sensitivity of 0.838 and a specificity of 0.827, better than lactate, which had a sensitivity of 0.892 and a specificity of 0.65 [55]. 

### 2.2. Cell-Free DNA

Recently, the mechanism of extensive cell death of immune cells induced by infection, which releases a large amount of damage-associated molecular patterns (DAMPs), triggers the host’s immune response, activates coagulation, and mediates multiple-organ dysfunction syndrome (MODS) [56,57,58,59]. Therefore, they play a central role in the development of sepsis and its progression to MODS [60,61].

DAMPs are endogenous molecules that activate the immune system. Examples of DAMPs include nuclear and mitochondrial DNA, RNA, nucleotides, heat shock proteins, and uric acid. These molecules are normally inside cells, performing different functions in homeostasis, but are released into the extracellular space when cells are exposed to stress. Cellular stress can be caused by various physical, chemical, metabolic, and infectious factors [62]. The mechanism of infection-induced extensive immune cell death releases a large quantity of DAMPs, which plays a role in the development of MODS and immunosuppression during sepsis [60,61,62,63]. Growing evidence supports the hypothesis that DAMPs, including high-mobility group protein (HMGB1), cell-free DNA (cfDNA), histones, and neutrophil extracellular traps (NETs), may directly or indirectly contribute significantly to the development of MODS [56].

Extracellular DNA, or cfDNA, is the DNA molecule released, whole or fragmented, into body fluids of healthy subjects, such as blood, saliva, urine and/or feces [64,65], from a nucleated cell, and its level is usually much higher in people after physical effort [65]; however, in patients with various diseases such as sepsis, the inflammatory stress significantly increases many markers as well as the cell-free DNA level [56,63,64,65,66,67,68,69]. Additionally, cf-DNA has been suggested as a potential predictive biomarker for several different conditions, including cancer (currently allows determination of a small fraction of tumor-derived) [70,71,72], stroke [73], trauma [74], transplantation (early detection of allograft rejection) [68,69,75,76], AKI [77,78], inflammatory and autoimmune diseases [79], and pregnancy (huge impact on prenatal medicine, where non-invasive prenatal testing is based on analysis of a fetal component of cfDNA in maternal blood) [80].

The majority of cfDNA in urine originates from plasma, but it can also arise from blood, kidney, and urinary tract cells. This fragment, retained in the glomeruli by mechanisms that are still unknown, is called transrenal cfDNA and has a lower concentration compared to other body fluids, which may also occur due to the greater enzymatic activity in the kidney than in other organs analyzed. However, when there is associated renal injury, such as carcinoma, infection, or transplantation, there is a greater and earlier release of the amount of fragmented mitochondrial and nuclear cfDNA in the urine, compared to other biochemical parameters, which can be exploited to detect early signs of severity [81]. Similar to plasma cfDNA, urinary cfDNA is nonspecific and can increase in several diseases; however, combining quantification and analysis with other parameters can increase its diagnostic value, like kidney injury molecule-1 and NGAL [81].

Clementi et al. demonstrated that, in septic patients who developed AKI, cfDNA levels were higher than in septic patients who did not develop AKI. In an animal model, total cfDNA increased in individuals with AKI, mainly influenced by the nuclear fraction, originating from the affected organ; however, the concentrations are dependent on the mechanism of injury induction and the animal model used [78].

In a separate investigation, cfDNA levels were assessed in an animal model with induced liver injury, revealing elevated cfDNA levels alongside other specific biomarkers. Nonetheless, this study acknowledges that cfDNA elevation post-tissue damage may limit its clinical utility and impact on prognostic evaluations [77].

Furthermore, a distinct study highlighted that heightened cfDNA levels served as a prognostic indicator for individuals with sepsis-induced acute kidney injury [81]. Benchtop in vivo experiments utilizing a CLP mouse model of sepsis indicated that increased cfDNA levels in the early sepsis phase could potentially function as a biomarker for assessing sepsis severity [69]. Subsequent clinical studies have corroborated the association between cfDNA levels and the clinical outcomes’ severity in sepsis cases [68,69].

A small study involving 34 patients, with 27 being septic and 7 non-septic, showed that cfDNA concentrations at admission were higher in non-survivors than in survivors [82]. In the retrospective study, stored plasma samples from 80 patients with severe sepsis were used to measure cfDNA, IL-6, thrombin, and protein. The levels of cfDNA in plasma had better prognostic utility than MODS or APACHE II scores, or the biomarkers measured by the AUC for cfDNA to predict ICU mortality is 0.97 (95% CI, 0.93 to 1.00) and to predict hospital mortality is 0.84 (95% CI, 0.75 to 0.94). They found that a cfDNA cut-off value of 2.35 ng/μL had a sensitivity of 87.9% and specificity of 93.5% for predicting ICU mortality [83]. A study carried out with cfDNA demonstrated that, in septic patients, those who presented profound damage to mitochondrial DNA and RNA oxidation had a greater association with long-term mortality [84].

The study by Long et al. used 78 plasma samples from ICU patients to compare next-generation sequencing (NGS) with traditional bacterial culture (BC) technology for pathogen identification from cfDNA plasma. They demonstrated levels of cfDNA that were twenty-two times higher compared to non-septic patients [85]. 

The mechanisms that modulate fibrinolysis in sepsis remain incomprehensible. Studies have revealed that high concentrations of endogenous cfDNA correlate with an increase in endogenous thrombin potential in plasmas obtained from patients with severe sepsis [86,87,88]. In addition to coagulation activation and negative regulation of endogenous anticoagulant pathways, dysfunction of the fibrinolytic system plays a central role in the pathogenesis of microvascular thrombosis and organ dysfunction in sepsis [87,88]. 

### 2.3. Proenkephalin—PENK

Proenkephalin (PENK) is filtrated by the glomerulus and is a newer biomarker of glomerular filtration. It has also been studied in sepsis settings, heart failure, CKD, and kidney transplants [89]. PENK is a stable surrogate marker for endogenous enkephalins and is a monomeric peptide with a molecular weight of approximately 4.5 kDa. It is cleaved from the precursor peptide pre-proenkephalin A alongside enkephalins, which are endogenous opioids that act primarily on delta opioid receptors. The first site with the highest density of delta opioid receptors is the central nervous system, and the second is the kidney [90].

The exact function of PENK is still unknown, but there is evidence to suggest that it may play a regulatory role in kidney function, such as inducing diuresis and natriuresis through receptor agonism or inhibiting antidiuretic hormone [91,92]. Plasma PENK concentration appears to correlate strongly with GFR [93]. Additionally, PENK has a long in vivo half-life, is stable after collection, is not influenced by age, sex, and inflammation, is not protein-bound in plasma, and is exclusively filtrated in the glomerulus, making it a very reliable indicator of glomerular filtration injury [94,95,96]. As a newer biomarker, there are limited studies, primarily focused on renal dysfunction and sepsis, but other research has found its association with heart failure and transplantation [97]. In a small cohort of liver transplant patients, PENK was found to be an independent predictor of severe AKI, with an AUC of 0.83 (CI 0.72–0.94), a cut-off of 119.05 pmol/L, a sensitivity of 0.81, a specificity of 0.90, and an accuracy of 0.84. It was also identified that PENK was 48 h earlier than serum creatinine in detecting severe AKI [88]. A recent meta-analysis, including 11 observational studies with 3969 patients, found the best optimal cut-off value of PENK for early detection of AKI to be 57.3 pmol/L. The sensitivity and specificity of PENK in identifying AKI were 0.69 (95% CI 0.62–0.75) and 0.76 (95% CI 0.68–0.82), respectively [98].

A study was conducted with 88 critically ill patients diagnosed with sepsis or septic shock. During admission to the Intensive Care Unit (ICU), the levels of serum creatinine and sroenkephalin were evaluated on days 0, 2, and 7. It was observed that patients who developed Acute Kidney Injury (AKI) showed a significant increase in proenkephalin levels in the plasma, reaching 145.0 pmol/l for septic AKI, with a sensitivity of 67.9% and specificity of 98.3%, and an area under the curve (AUC) of 0.796 [93].

Most studies involving PENK have focused on sepsis. In this context, three significant studies were carried out. The first study, which included 101 septic patients, revealed a correlation between PENK levels and the diagnosis and severity of acute kidney injury (AKI) based on the RIFLE criterion [99]. The second study, involving a cohort of 167 patients, found that PENK levels increased with the severity of sepsis and the diagnosis of AKI [100]. In the third study, which had a larger cohort of 978 patients, PENK was identified as a reliable indicator of AKI, the necessity for renal replacement therapy (RRT), and mortality, with odds ratios of 4.0 (95% CI 3.0–5.4) and 1.5 (95% CI 1.2–1.8), respectively [101]. A review demonstrated that increased plasma PENK concentration is directly related to the evolution of kidney disease and long-term mortality, being a potential surrogate to estimate renal deterioration [93].

### 2.4. Cut-Off Values Biomarkers

The cut-off values play a crucial role in the diagnosis of acute kidney injury (AKI) and sepsis, guiding clinicians in distinguishing between these conditions. Lower cut-off values are often observed in AKI, while higher values are common in sepsis diagnosis. Typically, studies focus on only one pathology or subpathology, such as AKI related to sepsis. However, taking a broad approach that encompasses both conditions through the same biomarker can enhance the understanding of the crosstalk between them, identify therapeutic targets, and prognostics, and comprehend the progression to CKD severity in AKI and septic shock in sepsis. Table 3 presents the optimal cut-off values for each biomarker in diagnosing sepsis and acute kidney injury (AKI), along with their corresponding sensitivities and specificities [102].

There are other biomarkers involved in the diagnosis of sepsis, one of them being neutrophil extracellular traps (NETs), discovered in 2004 [103]. Their increased presence is observed in many inflammatory and autoimmune diseases. In sepsis scenarios, NETs act as the “first line” of defense against pathogens at the site of inflammation, preventing the spread of infection from the inflammatory focus [104]. This contributes to the subsequent destruction of pathogens by antimicrobial proteins and professional phagocytes in the early stages of sepsis, showing bactericidal effects. However, as the disease progresses, NETs can lead to damage in the lungs and liver [105,106,107].

## 3. Conclusions

The diagnosis of sepsis and AKI poses a significant challenge in clinical practice, with delayed diagnosis linked to unfavorable outcomes. The pathophysiological interplay between these conditions underscores the need for more advanced diagnostic approaches. NGAL, for instance, has shown efficacy in identifying septic patients and predicting disease severity, while cfDNA has been linked to the severity of clinical outcomes in sepsis. Furthermore, PENK has emerged as a reliable indicator of glomerular filtration injury and has been associated with the severity of AKI in septic patients. Biomarkers such as NGAL, PENK, and cfDNA not only have the potential to elucidate this complex relationship pathophysiological but also to enhance risk stratification and diagnostic accuracy for these pathologies in the future.

## 4. Future Directions

The article proposes the evaluation of AKI and sepsis by the same biomarker. Firstly, both are considered confounding factors: sepsis can lead to AKI, and AKI can be associated with sepsis. By conducting a joint evaluation, a more comprehensive interpretation can be achieved. 

The largest number of studies was carried out with NGAL, but they are still insufficient to determine the cost–benefit of its application. In some scenarios, participants had more serious conditions that required standard care, so it was not possible to exclusively determine the cost-effectiveness of the biomarker [108]. Another study highlights the lack of clinical trials on the impact of biomarkers on direct health outcomes, such as the need for ICU, progression to CKD, and mortality prediction, which makes it impossible to measure the advantages between effective costs and their effects. It is important to highlight those critically ill patients who require intensive care and prolonged hospitalizations, are more prone to complications, leading to a greater burden on health services [52]. Therefore, once the effectiveness of these biomarkers has been proven, their final benefits will initially justify their values. Furthermore, more studies are needed that explore cfDNA and PENK from this perspective.

Future research in the field of sepsis and AKI diagnosis is driven by the development and validation of biomarkers. Biomarkers such as NGAL, cell-free DNA, and PENK are emerging as promising tools to enhance early detection, risk stratification, and prognosis of these conditions. Understanding the biomarker kinetics is crucial, especially in sepsis, where the diagnosis is commonly made in patients with "suspected sepsis," which may not indicate early diagnosis as the condition may already be established. Few studies on AKI use the pre-pathological phase to determine the most realistic scenario of early detection compared to conventional diagnosis and thus diagnose subclinical AKI that may respond better to interventions than functional AKI. Therefore, studies should further explore the main limitations encountered: cut-off values, prognostic assessment, cost-effectiveness relationship, understanding of kinetics, and early diagnostic accuracy.

## Figures and Tables

**Table 1:** Classification of Acute Kidney Injury.

Stages	Serum Creatinine	Urine Output
Stage 1	Creatinine above 1.5–1.9 times (compared to baseline) in 7 days or increase of 0.3 mg/dL (>26.5 micromol/L) in 48 h	Less than 0.5 mL/kg/h for 6 to 12 h
Stage 2	Increase greater than 2.0–2.9 times (compared to baseline)	Less than 0.5 mL/kg/h for more than 12 h
Stage 3	Increase greater than 3.0 times the baseline value or creatinine above 4.0 mg/dL	Less than 0.3 mg/kg/h for 24 h or anuria for more than 12 h

**Table 2:** Pathophysiology in the diagnosis of AKI and sepsis and confounding factors of the biomarkers NGAL, PENK, and cfDNA.

	Diagnosis of AKI	Diagnosis Sepsis	Confounding Factors
NGAL	Is filtered by the glomerulus and reabsorbed by the proximal tubule, the increased expression in urine or plasma is associated with AKI.	In the severe systemic inflammatory response that accompanies sepsis, the homodimer type of NGAL released by neutrophils is released.	Chronic obstructive pulmonary disease, cardiac dysfunction, hypertension, and diabetes.
cfDNA	cfDNA in urine originates from plasma, where it fragments, being retained in the glomeruli by transrenal cfDNA. In cases of associated renal injury, such as carcinoma, infection, or transplantation, there is a greater and earlier release of fragmented mitochondrial and nuclear cfDNA in the urine.	Cellular stress induced by infectious factors leads to cell death and the release of a large quantity of cfDNA, which contributes to the progression of multiple-organ dysfunction syndrome.	Cancer, stroke, trauma, transplantation, pregnancy, physical effort, inflammatory and autoimmune diseases.
PENK	The kidney is the second organ in the body with delta opioid receptors. PENK is exclusively filtered in the glomerulus and plays a role in inducing diuresis and natriuresis through receptor agonism or by inhibiting antidiuretic hormone. It is strongly correlated with GFR and the progression of AKI.	During sepsis, the pathophysiology of PENK involves modulating the inflammatory response, which includes the release of pro-inflammatory cytokines and the activation of neuro-humoral pathways.	Heart failure and transplant.

Legend: acute kidney injury (AKI), neutrophil gelatinase-associated lipocalin (NGAL), cell-free DNA (cfDNA), proenkephalin (PENK), glomerular filtration rate (GFR).

**Table 3:** Cut-off value and area under the curve of the biomarkers NGAL, cfDNA, and PENK in selected studies.

Cut-Off Value	AKI	Se	Sp	AUC	Sepsis	Se	Sp	AUC	Ref. AKI and Sepsis
NGAL (ng/mL)	150	82%	97%	0.81	250	83%	82%	0.69	46, 50
cfDNA (ng/mL)	161.3	67%	92%	0.82	1.52	83%	79%	0.97	80, 78
PENK (pmol/L)	119	81%	90%	0.83	145	67.9%	98%	0.79	92, 106

Legend: acute kidney injury (AKI), neutrophil gelatinase-associated lipocalin (NGAL), cell-free DNA (cfDNA), proenkephalin (PENK), sensitivity (Se), specificity (Sp), area under curve (AUC), Ref. (reference).

## Data Availability

No new data were created or analyzed in this study. Data sharing is not applicable to this article.

## References

[1] Solomon R., Ferreira B.L., Solomon M.C., Santos S.S., Azevedo L.C.P., Brunialti M.K.C. (2019). Sepsis: Evolving concepts and challenges. Braz. J. Med. Biol. Res..

[2] Napolitano L.M. (2018). Sepsis 2018: Definitions and Guideline Changes. Surg. Infect..

[3] WHO (World Health Organization) (2017). Improving the prevention, diagnosis and clinical management of sepsis. Rep. Secr..

[4] Evans L., Rhodes A., Alhazzani W., Antonelli M., Coopersmith C.M., French C., Machado F.R., Mcintyre L., Ostermann M., Prescott H.C. (2021). Surviving sepsis campaign: International guidelines for management of sepsis and septic shock 2021. Intensive Care Med..

[5] Fleischmann-Struzek C., Mellhammar L., Rose N., Cassini A., Rudd K.E., Schlattmann P., Allegranzi B., Reinhart K. (2020). Incidence and mortality of hospital- and ICU-treated sepsis: Results from an updated and expanded systematic review and meta-analysis. Intensive Care Med..

[6] Font M.D., Thyagarajan B., Khanna A.K. (2020). Sepsis and Septic Shock—Basics of diagnosis, pathophysiology and clinical decision making. Med. Clin. N. Am..

[7] Ostermann M., Cerdá J. (2018). The Burden of Acute Kidney Injury and Related Financial Issues. Contrib. Nephrol..

[8] Kellum J.A., Romagnani P., Ashuntantang G., Ronco C., Zarbock A., Anders H.-J. (2021). Acute kidney injury. Nat. Rev. Dis. Prim..

[9] Pickkers P., Darmon M., Hoste E., Joannidis M., Legrand M., Ostermann M., Prowle J.R., Schneider A., Schetz M. (2021). Acute kidney injury in the critically ill: An updated review on pathophysiology and management. Intensive Care Med..

[10] Fang Y., Ding X., Zhong Y., Zou J., Teng J., Tang Y., Lin J., Lin P. (2010). Acute kidney injury in a Chinese hospitalized population. Blood Purif..

[11] Hsu C.-Y., McCulloch C., Fan D., Ordoñez J., Chertow G., Go A. (2007). Community-based incidence of acute renal failure. Kidney Int..

[12] Lewington A.J., Cerdá J., Mehta R.L. (2013). Raising awareness of acute kidney injury: A global perspective of a silent killer. Kidney Int..

[13] Uchino S., Kellum J.A., Bellomo R., Doig G.S., Morimatsu H., Morgera S., Schetz M., Tan I., Bouman C., Macedo E. (2005). Acute renal failure in critically ill patients: A multinational, multicenter study. JAMA.

[14] Murugan R., Kellum J.A. (2011). Acute kidney injury: What’s the prognosis?. Nat. Rev. Nephrol..

[15] Jacobi J. (2022). The pathophysiology of sepsis—2021 update: Part 2, organ dysfunction and assessment. Am. J. Health-Syst. Pharm..

[16] Gameiro J., Fonseca J.A., Jorge S., Lopes J.A. (2018). Acute Kidney Injury Definition and Diagnosis: A Narrative Review. J. Clin. Med..

[17] Bone R.C., Balk R.A., Cerra F.B., Dellinger R.P., Fein A.M., Knaus W.A., Schein R.M.H., Sibbald W.J. (1992). Definitions for sepsis and organ failure and guidelines for the use of innovative therapies in sepsis. The ACCP/SCCM Consensus Conference Committee. American College of Chest Physicians/Society of Critical Care Medicine. Chest.

[18] Levy M.M., Fink M.P., Marshall J.C., Abraham E., Angus D., Cook D., Cohen J., Opal S.M., Vincent J.-L., Ramsay G. (2003). 2001 SCCM/ESICM/ACCP/ATS/SIS International Sepsis Definitions Conference. Crit. Care Med..

[19] Seymour C.W., Liu V.X., Iwashyna T.J., Brunkhorst F.M., Rea T.D., Scherag A., Rubenfeld G., Kahn J.M., Shankar-Hari M., Singer M. (2016). Assessment of Clinical Criteria for Sepsis: For the Third International Consensus Definitions for Sepsis and Septic Shock (Sepsis-3). JAMA.

[20] Shankar-Hari M., Phillips G.S., Levy M.L., Seymour C.W., Liu V.X., Deutschman C.S., Angus D.C., Rubenfeld G.D., Singer M. (2016). Developing a New Definition and Assessing New Clinical Criteria for Septic Shock: For the Third International Consensus Definitions for Sepsis and Septic Shock (Sepsis-3). JAMA.

[21] Singer M., Deutschman C.S., Seymour C.W., Shankar-Hari M., Annane D., Bauer M., Bellomo R., Bernard G.R., Chiche J.-D., Coopersmith C.M. (2016). The Third International Consensus Definitions for Sepsis and Septic Shock (Sepsis-3). JAMA.

[22] Doualeh M., Payne M., Litton E., Raby E., Currie A. (2022). Molecular Methodologies for Improved Polymicrobial Sepsis Diagnosis. Int. J. Mol. Sci..

[23] Khwaja A. (2012). KDIGO Clinical practice guidelines for acute kidney injury. Nephron Clin. Pr..

[24] Hoste E.A.J., Bagshaw S.M., Bellomo R., Cely C.M., Colman R., Cruz D.N., Edipidis K., Forni L.G., Gomersall C.D., Govil D. (2015). Epidemiology of acute kidney injury in critically ill patients: The multinational AKI-EPI study. Intensive Care Med..

[25] Zarbock A., Nadim M.K., Pickkers P., Gomez H., Bell S., Joannidis M., Kashani K., Koyner J.L., Pannu N., Meersch M. (2023). Sepsis-associated acute kidney injury: Consensus report of the 28th Acute Disease Quality Initiative workgroup. Nat. Rev. Nephrol..

[26] Zatz R., Seguro A.C., Malnic G. (2011). Physiological Bases of Nephrology.

[27] Machado F.R., Cavalcanti A.B., Bozza F.A., Ferreira E.M., Angotti Carrara F.S., Sousa J.L., Caixeta N., Salomao R., Angus D.C., Pontes Azevedo L.C. (2017). The epidemiology of sepsis in Brazilian intensive care units (the Sepsis PREvalence Assessment Database, SPREAD): An observational study. Lancet Infect. Dis..

[28] Pinheiro K.H.E., Azêdo F.A., Areco K.C.N., Laranja S.M.R. (2019). Risk factors and mortality in patients with sepsis, septic and non septic acute kidney injury in ICU. Braz. J. Nephrol..

[29] Barbosa J.S., Silva Júnior G.B., Meneses G.C., Martins A.M.C., Daher E.D.F., Machado R.P.G., Rudders R.P.G. (2022). Non-traditional biomarkers of acute kidney injury in premature newborns with sepsis: Early diagnosis. Braz. J. Nephrol..

[30] Lima C., Macedo E. (2018). Urinary Biochemistry in the Diagnosis of Acute Kidney Injury. Dis. Markers.

[31] Samsudin I., Vasikaran S.D. (2017). Clinical Utility and Measurement of Procalcitonin. Clin. Biochem. Rev..

[32] Meisner M. (2014). Update on Procalcitonin Measurements. Ann. Lab. Med..

[33] Clec’h C., Ferriere F., Karoubi P., Fosse J.P., Cupa M., Hoang P., Cohen Y. (2004). Diagnostic and prognostic value of procalcitonin in patients with septic shock. Crit. Care Med..

[34] Kjeldsen L., Johnsen A.H., Sengeløv H., Borregaard N. (1993). Isolation and primary structure of NGAL, a novel protein associated with human neutrophil gelatinase. J. Biol. Chem..

[35] Cowland J.B., Borregaard N. (1997). Molecular characterization and pattern of tissue expression of the gene for neutrophil gelatinase-associated lipocalin from humans. Genomics.

[36] Nielsen B.S., Borregaard N., Bundgaard J.R., Timshel S., Sehested M., Kjeldsen L. (1996). Induction of NGAL synthesis in epithelial cells of human colorectal neoplasia and inflammatory bowel diseases. Gut.

[37] Hvidberg V., Jacobsen C., Strong R.K., Cowland J.B., Moestrup S.K., Borregaard N. (2005). The endocytic receptor megalin binds the iron transporting neutrophil-gelatinase-associated lipocalin with high affinity and mediates its cellular uptake. FEBS Lett..

[38] Schmidt-Ott K.M., Chen X., Paragas N., Levinson R.S., Mendelsohn C.L., Barasch J. (2006). c-kit delineates a distinct domain of progenitors in the developing kidney. Dev. Biol..

[39] Mori K., Lee H.T., Rapoport D., Drexler I.R., Foster K., Yang J., Schmidt-Ott K.M., Chen X., Li J.Y., Weiss S. (2005). Endocytic delivery of lipocalin-siderophore-iron complex rescues the kidney from ischemia-reperfusion injury. J. Clin. Investig..

[40] Yang J., Goetz D., Li J.-Y., Wang W., Mori K., Setlik D., Du T., Erdjument-Bromage H., Tempst P., Strong R. (2002). An Iron Delivery Pathway Mediated by a Lipocalin. Mol. Cell.

[41] Marakala V. (2022). Neutrophil gelatinase-associated lipocalin (NGAL) in kidney injury—A systematic review. Clin. Chim. Acta.

[42] Legrand M., Darmon M., Joannidis M. (2013). NGAL and AKI: The end of a myth?. Intensive Care Med..

[43] Cullen M.R., Murray P.T., Fitzgibbon M.C. (2012). Establishment of a reference interval for urinary neutrophil gelatinase-associated lipocalin. Ann. Clin. Biochem. Int. J. Biochem. Lab. Med..

[44] Pennemans V., Rigo J.-M., Faes C., Reynders C., Penders J., Swennen Q. (2013). Establishment of reference values for novel urinary biomarkers for renal damage in the healthy population: Are age and gender an issue?. Clin. Chem. Lab. Med..

[45] Lima C., de Fatima Vattimo M., Macedo E. (2022). Neutrophil Gelatinase-Associated Lipocalin as a Promising Biomarker in Acute Kidney Injury. Inflammation in the 21st Century.

[46] Singer E., Elger A., Elitok S., Kettritz R., Nickolas T.L., Barasch J., Luft F.C., Schmidt-Ott K.M. (2011). Urinary neutrophil gelatinase-associated lipocalin distinguishes pre-renal from intrinsic renal failure and predicts outcomes. Kidney Int..

[47] Zhou F., Luo Q., Wang L., Han L. (2015). Diagnostic value of neutrophil gelatinase-associated lipocalin for early diagnosis of cardiac surgery-associated acute kidney injury: A meta-analysis. Eur. J. Cardio-Thorac. Surg..

[48] Constantin J.-M., Futier E., Perbet S., Roszyk L., Lautrette A., Gillart T., Guerin R., Jabaudon M., Souweine B., Bazin J.-E. (2010). Plasma neutrophil gelatinase-associated lipocalin is an early marker of acute kidney injury in adult critically ill patients: A prospective study. J. Crit. Care.

[49] Di Somma S., Magrini L., De Berardinis B., Marino R., Ferri E., Moscatelli P., Ballarino P., Carpinteri G., Noto P., Gliozzo B. (2013). Additive value of blood neutrophil gelatinase-associated lipocalin to clinical judgement in acute kidney injury diagnosis and mortality prediction in patients hospitalized from the emergency department. Crit. Care.

[50] Haase M., Bellomo R., Devarajan P., Schlattmann P., Haase-Fielitz A., NGAL Meta-Analysis Investigator Group (2009). Accuracy of Neutrophil Gelatinase-Associated Lipocalin (NGAL) in Diagnosis and Prognosis in Acute Kidney Injury: A Systematic Review and Meta-analysis. Am. J. Kidney Dis..

[51] Liu Y., Bu L., Chao Y., Wang H. (2022). Combined Serum NGAL and Fetuin-A to Predict 28-Day Mortality in Patients with Sepsis and Risk Prediction Model Construction. Cell. Mol. Biol..

[52] Jacobsen E., Sawhney S., Brazzelli M., Aucott L., Scotland G., Aceves-Martins M., Robertson C., Imamura M., Poobalan A., Manson P. (2021). Cost-effectiveness and value of information analysis of NephroCheck and NGAL tests compared to standard care for the diagnosis of acute kidney injury. BMC Nephrol..

[53] Gowri M., Iyyadurai R., Abhilash K.P.P., Paul A., Newbigging N.S., Lenin A., Varghese J.S., Nell A.J., Binu A.J., Chandiraseharan V.K. (2023). Role of Neutrophil Gelatinase-associated Lipocalin (NGAL) and Other Clinical Parameters as Predictors of Bacterial Sepsis in Patients Presenting to the Emergency Department with Fever. Indian J. Crit. Care Med..

[54] Laurberg M., Saegerman C., Jacobsen S., Berg L.C., Laursen S.H., Hoeberg E., Sånge E.A., van Galen G. (2023). Use of admission serum neutrophil gelatinase-associated lipocalin (NGAL) concentrations as a marker of sepsis and outcome in neonatal foals. PLoS ONE.

[55] Wu Y., Yu C., Zhou Y., He Z.-M., Zhang W., Fan J., Sun Y. (2022). Risk stratification and prognostic value of serum neutrophil gelatinase-associated lipocalin (sNGAL) in sepsis patients. Acta Biochim. Pol..

[56] Cheng Z., Abrams S.T., Toh J., Wang S.S., Wang Z., Yu Q., Yu W., Toh C.-H., Wang G. (2020). The Critical Roles and Mechanisms of Immune Cell Death in Sepsis. Front. Immunol..

[57] Cheng Z., Abrams S.T., Alhamdi Y., Toh J., Yu W., Wang G., Toh C.-H. (2019). Circulating Histones Are Major Mediators of Multiple Organ Dysfunction Syndrome in Acute Critical Illnesses. Crit. Care Med..

[58] Alhamdi Y., Abrams S.T., Cheng Z., Jing S., Su D., Liu Z., Lane S., Welters I., Wang G., Toh C.-H. (2015). Circulating Histones Are Major Mediators of Cardiac Injury in Patients with Sepsis. Crit. Care Med..

[59] Abrams S.T., Zhang N., Manson J., Liu T., Dart C., Baluwa F., Wang S.S., Brohi K., Kipar A., Yu W. (2013). Circulating histones are mediators of trauma-associated lung injury. Am. J. Respir. Crit. Care Med..

[60] Matzinger P. (2002). The Danger Model: A Renewed Sense of Self. Science.

[61] Gentile L.F., Moldawer L.L. (2013). DAMPs, PAMPs, and the Origins of SIRS in Bacterial Sepsis. Shock.

[62] Murao A., Aziz M., Wang H., Brenner M., Wang P. (2021). Release mechanisms of major DAMPs. Apoptosis.

[63] Rajaee A., Barnett R., Cheadle W.G. (2018). Pathogen- and Danger-Associated Molecular Patterns and the Cytokine Response in Sepsis. Surg. Infect..

[64] Pisetsky D.S. (2012). The origin and properties of extracellular DNA: From PAMP to DAMP. Clin. Immunol..

[65] Bronkhorst A.J., Ungerer V., Oberhofer A., Gabriel S., Polatoglou E., Randeu H., Uhlig C., Pfister H., Mayer Z., Holdenrieder S. (2022). New Perspectives on the Importance of Cell-Free DNA Biology. Diagnostics.

[66] Stawski R., Stec-Martyna E., Chmielecki A., Nowak D., Perdas E. (2021). Current trends in cell-free DNA applications. scoping review of clinical trials. Biology.

[67] Hamaguchi S., Akeda Y., Yamamoto N., Seki M., Yamamoto K., Oishi K., Tomono K. (2015). Origin of Circulating Free DNA in Sepsis: Analysis of the CLP Mouse Model. Mediat. Inflamm..

[68] Margraf S., Lögters T., Reipen J., Altrichter J., Scholz M., Windolf J. (2008). neutrophil-derived circulating free DNA (cf-DNA/NETs): A potential prognostic marker for posttraumatic development of inflammatory second hit and sepsis. Shock.

[69] Lögters T., Paunel-Görgülü A., Zilkens C., Altrichter J., Scholz M., Thelen S., Krauspe R., Margraf S., Jeri T., Windolf J. (2009). Diagnostic accuracy of neutrophil-derived circulating free DNA (cf-DNA/NETs) for septic arthritis. J. Orthop. Res..

[70] Wei L., Xie L., Wang X., Ma H., Lv L., Liu L., Song X. (2018). Circulating tumor DNA measurement provides reliable mutation detection in mice with human lung cancer xenografts. Lab. Investig..

[71] Yangyanqiu W., Shuwen H. (2022). Bacterial DNA involvement in carcinogenesis. Front. Cell. Infect. Microbiol..

[72] Swystun L.L., Mukherjee S., Liaw P.C. (2011). Breast cancer chemotherapy induces the release of cell-free DNA, a novel procoagulant stimulus. J. Thromb. Haemost..

[73] Rainer T.H., Wong L.K., Lam W., Yuen E., Lam N.Y., Metreweli C., Lo Y.D. (2003). Prognostic use of circulating plasma nucleic acid concentrations in patients with acute stroke. Clin. Chem..

[74] Lo Y.M.D., Rainer T.H., Chan L.Y.S., Hjelm N.M., A Cocks R. (2000). Plasma DNA as a Prognostic Marker in Trauma Patients. Clin. Chem..

[75] Sherwood K., Weimer E.T. (2018). Characteristics, properties, and potential applications of circulating cell-free DNA in clinical diagnostics: A focus on transplantation. J. Immunol. Methods.

[76] Oellerich M., Budde K., Osmanodja B., Bornemann-Kolatzki K., Beck J., Schütz E., Walson P.D. (2022). Donor-derived cell-free DNA as a diagnostic tool in transplantation. Front. Genet..

[77] Gruenbaum B.F., Boyko M., Delgado B., Douvdevany A., Gruenbaum S.E., Melamed I., Gideon M., Cesnulis E., Shapira Y., Zlotnik A. (2012). Cell-free DNA as a potential marker to predict carbon tetrachloride-induced acute liver injury in rats. Hepatol. Int..

[78] Homolová J., Janovičová L., Konečná B., Vlková B., Celec P., Tóthová L., Bábíčková J. (2020). Plasma Concentrations of Extracellular DNA in Acute Kidney Injury. Diagnostics.

[79] Mondelo-Macía P., Castro-Santos P., Castillo-García A., Muinelo-Romay L., Diaz-Peña R. (2021). Circulating Free DNA and Its Emerging Role in Autoimmune Diseases. J. Pers. Med..

[80] Swarup V., Rajeswari M. (2007). Circulating (cell-free) nucleic acids—A promising, non-invasive tool for early detection of several human diseases. FEBS Lett..

[81] Celec P., Vlková B., Lauková L., Bábíčková J., Boor P. (2018). Cell-free DNA: The role in pathophysiology and as a biomarker in kidney diseases. Expert Rev. Mol. Med..

[82] Clementi A., Virzì G.M., Brocca A., Pastori S., de Cal M., Marcante S., Granata A., Ronco C. (2016). The Role of Cell-Free Plasma DNA in Critically Ill Patients with Sepsis. Blood Purif..

[83] Dwivedi D.J., Toltl L.J., Swystun L.L., Pogue J., Liaw K.-L., I Weitz J., Cook D.J., E Fox-Robichaud A., Liaw P.C. (2012). Prognostic utility and characterization of cell-free DNA in patients with severe sepsis. Crit. Care.

[84] van der Slikke E.C., Star B.S., Quinten V.M., ter Maaten J.C., Ligtenberg J.J., van Meurs M., Gansevoort R.T., Bakker S.J., Chao M.-R., Henning R.H. (2022). Association between oxidized nucleobases and mitochondrial DNA damage with long-term mortality in patients with sepsis. Free Radic. Biol. Med..

[85] Long Y., Zhang Y., Gong Y., Sun R., Su L., Lin X., Shen A., Zhou J., Caiji Z., Wang X. (2016). Diagnosis of Sepsis with Cell-free DNA by Next-Generation Sequencing Technology in ICU Patients. Arch. Med. Res..

[86] Gould T.J., Vu T.T., Stafford A.R., Dwivedi D.J., Kim P.Y., Fox-Robichaud A.E., Weitz J.I., Liaw P.C. (2015). Cell-Free DNA Modulates Clot Structure and Impairs Fibrinolysis in Sepsis. Arterioscler. Thromb. Vasc. Biol..

[87] Levi M., de Jonge E., van der Poll T. (2003). Sepsis and Disseminated Intravascular Coagulation. J. Thromb. Thrombolysis.

[88] Gando S. (2010). Microvascular thrombosis and multiple organ dysfunction syndrome. Crit. Care Med..

[89] Rosenqvist M., Bronton K., Hartmann O., Bergmann A., Struck J., Melander O. (2019). Proenkephalin a 119–159 (penKid)—A novel biomarker for acute kidney injury in sepsis: An observational study. BMC Emerg. Med..

[90] Denning G.M., Ackermann L.W., Barna T.J., Armstrong J.G., Stoll L.L., Weintraub N.L., Dickson E.W. (2008). Proenkephalin expression and enkephalin release are widely observed in non-neuronal tissues. Peptides.

[91] Sezen S.F., A Kenigs V., Kapusta D.R. (1998). Renal excretory responses produced by the delta opioid agonist, BW373U86, in conscious rats. Lancet.

[92] Grossman A., Besser G.M., Miles J.J., Baylis P.H. (1980). Inhibition of vasopressin release in man by an opioid peptide. Lancet.

[93] Khorashadi M., Beunders R., Pickkers P., Legrand M. (2020). Proenkephalin: A New Biomarker for Glomerular Filtration Rate and Acute Kidney Injury. Nephron.

[94] Donato L.J., Meeusen J.W., Lieske J.C., Bergmann D., Sparwaßer A., Jaffe A.S. (2018). Analytical performance of an immunoassay to measure proenkephalin. Clin. Biochem..

[95] Caironi P., Latini R., Struck J., Hartmann O., Bergmann A., Bellato V., Ferraris S., Tognoni G., Pesenti A., Gattinoni L. (2018). Circulating Proenkephalin, Acute Kidney Injury, and Its Improvement in Patients with Severe Sepsis or Shock. Clin. Chem..

[96] Slominski A.T., Zmijewski M.A., Zbytek B., Brozyna A.A., Granese J., Pisarchik A., Szczesniewski A., Tobin D.J. (2011). Regulated proenkephalin expression in human skin and cultured skin cells. J. Investig. Dermatol..

[97] Lima C., Gorab D.L., Fernandes C.R., Macedo E. (2022). Role of proenkephalin in the diagnosis of severe and subclinical acute kidney injury during the perioperative period of liver transplantation. Pract. Lab. Med..

[98] Lin L.-C., Chuan M.-H., Liu J.-H., Liao H.-W., Ng L.L., Magnusson M., Jujic A., Pan H.-C., Wu V.-C., Forni L.G. (2023). Proenkephalin as a biomarker correlates with acute kidney injury: A systematic review with meta-analysis and trial sequential analysis. Crit. Care.

[99] Marino R., Struck J., Hartmann O., Maisel A.S., Rehfeldt M., Magrini L., Melander O., Bergmann A., Di Somma S. (2015). Diagnostic and short-term prognostic utility of plasma pro-enkephalin (pro-ENK) for acute kidney injury in patients admitted with sepsis in the emergency department. J. Nephrol..

[100] Kim H., Hur M., Lee S., Marino R., Magrini L., Cardelli P., Struck J., Bergmann A., Hartmann O., Di Somma S. (2017). Proenkephalin, Neutrophil Gelatinase-Associated Lipocalin, and Estimated Glomerular Filtration Rates in Patients with Sepsis. Ann. Lab. Med..

[101] Hollinger A., Wittebole X., François B., Pickkers P., Antonelli M., Gayat E., Chousterman B.G., Lascarrou J.-B., Dugernier T., Di Somma S. (2018). Proenkephalin A 119-159 (Penkid) Is an Early Biomarker of Septic Acute Kidney Injury: The Kidney in Sepsis and Septic Shock (Kid-SSS) Study. Kidney Int. Rep..

[102] Hassan M.M., Arnob A.S., Ahmed A.H.H., Rahman A.K.M.S., Akbar A.A.G., Jabin P., Khan S.B., Singha A.K., Tahora S., Karim A.N.M.E. (2021). Proenkephalin is an Early Biomarker to Predict Septic Acute Kidney Injury among Patients in Intensive Care Unit. Arch. Nephrol. Urol..

[103] Brinkmann V., Reichard U., Goosmann C., Fauler B., Uhlemann Y., Weiss D.S., Weinrauch Y., Zychlinsky A. (2004). Neutrophil extracellular traps kill bacteria. Science.

[104] Vorobjeva N.V. (2020). Neutrophil Extracellular Traps: New Aspects. Mosc. Univ. Biol. Sci. Bull..

[105] Meng W., Paunel-Görgülü A., Flohé S., Hoffmann A., Witte I., MacKenzie C., E Baldus S., Windolf J., Lögters T.T. (2012). Depletion of neutrophil extracellular traps in vivo results in hypersusceptibility to polymicrobial sepsis in mice. Crit. Care.

[106] Weber C. (2015). Neutrophil extracellular traps mediate bacterial liver damage. Nat. Rev. Gastroenterol. Hepatol..

[107] Saffarzadeh M., Juenemann C., Queisser M.A., Lochnit G., Barreto G., Galuska S.P., Lohmeyer J., Preissner K.T. (2012). Neutrophil extracellular traps directly induce epithelial and endothelial cell death: A predominant role of histones. PLoS ONE.

[108] Brazzelli M., Aucott L., Aceves-Martins M., Robertson C., Jacobsen E., Imamura M., Poobalan A., Manson P., Scotland G., Kaye C. (2022). Biomarkers for assessing acute kidney injury for people who are being considered for admission to critical care: A systematic review and cost-effectiveness analysis. Health Technol. Assess..

